# Supplementary material for: DMRforPairs: identifying Differentially Methylated Regions between unique samples using array based methylation profiles
Source: BMC Bioinformatics. 2014 May 15;15:141. doi: 10.1186/1471-2105-15-141 (PMC4046028; doi:10.1186/1471-2105-15-141)
Supplement: Additional file 2 — DMRforPairs output for the comparison of A431-MCF7 and NA17018-NA17105. Please start from the HTML files in each folder. Available via the BMC Bioinformatics website. [file 1471-2105-15-141-S2.zip › 1394847754114233_MOESM2_ESM/A431_MCF7/figures/9784.pdf]

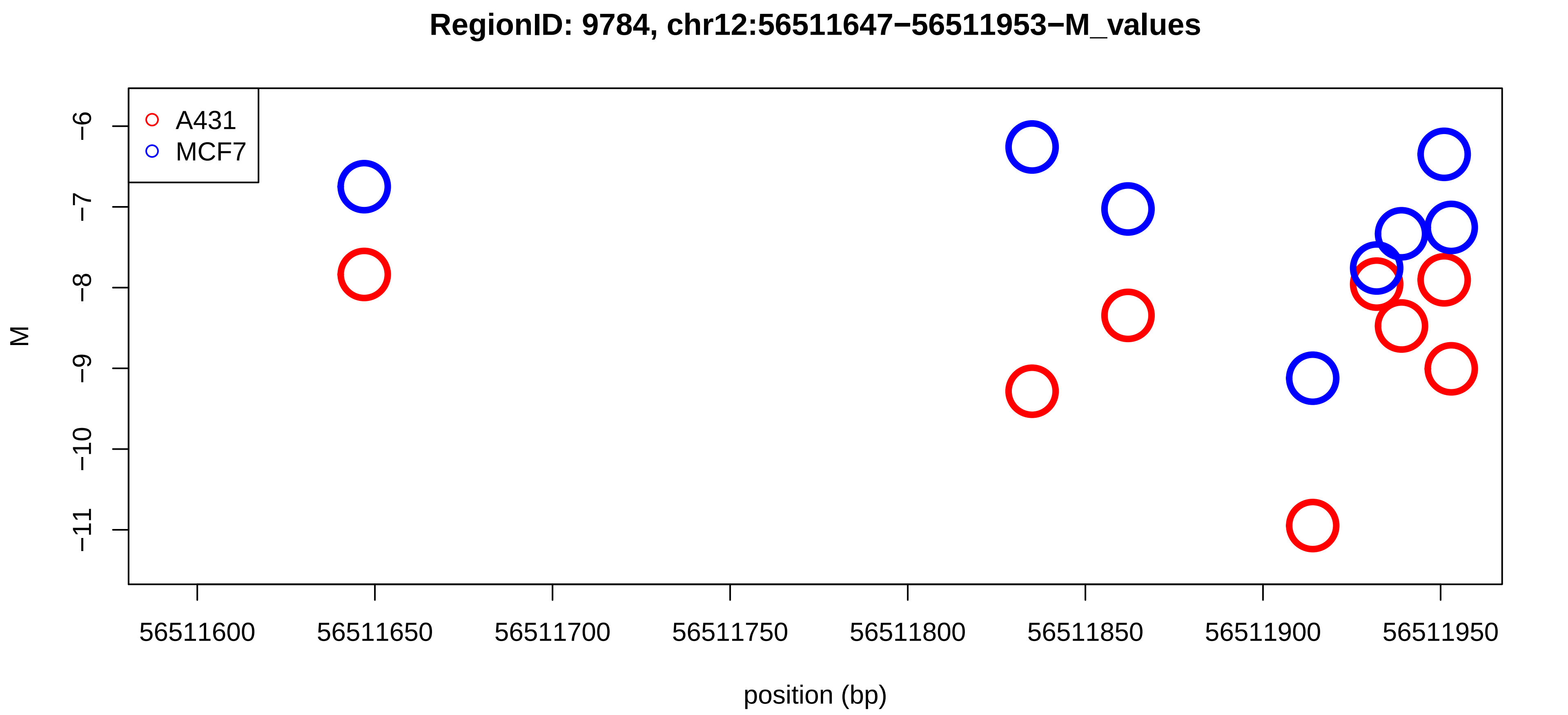
RegionID: 9784, chr12:56511647−56511953−M_values
A431
MCF7
M
−6
−7
−8
−9
−10
−11
56511600
56511650
56511700
56511750
56511800
56511850
56511900
56511950
position (bp)

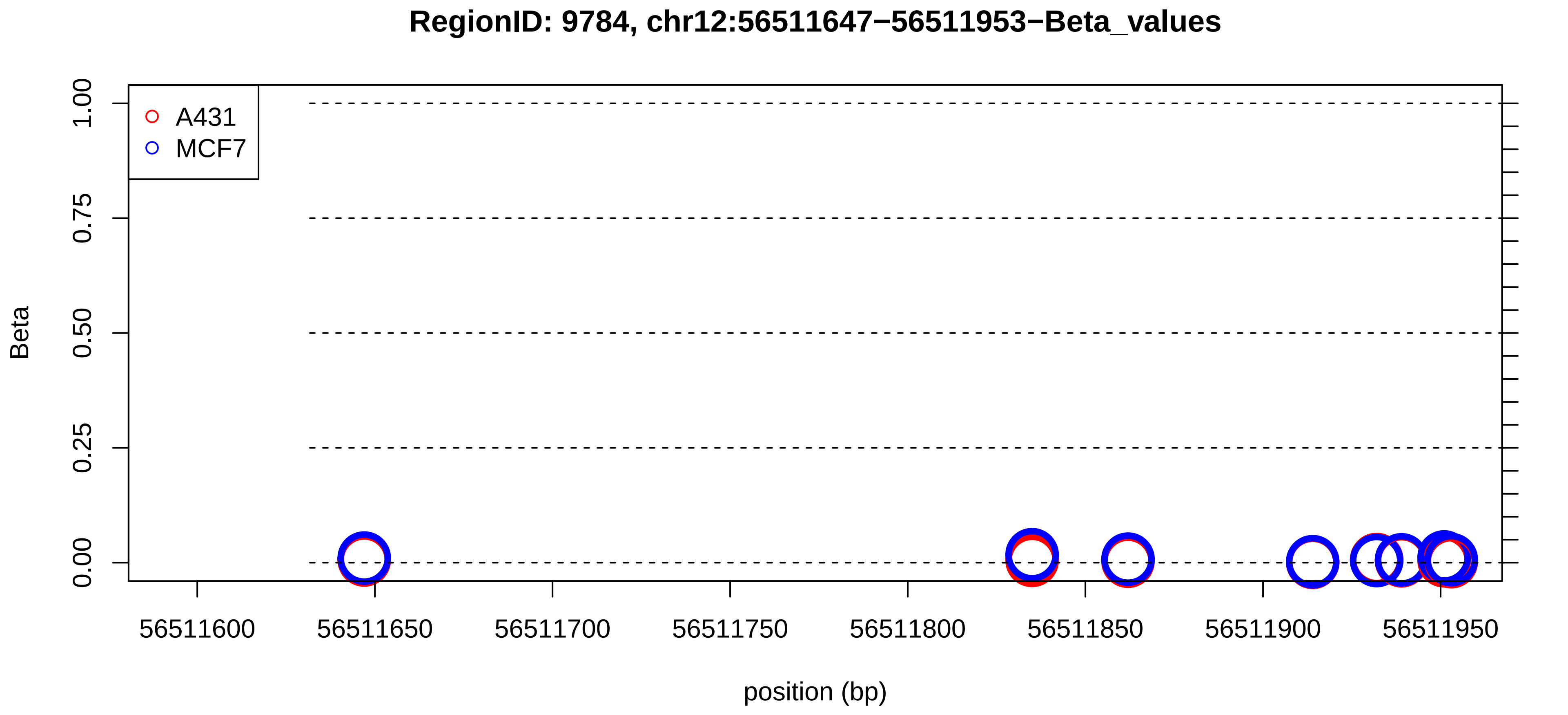
RegionID: 9784, chr12:56511647−56511953−Beta_values
A431
MCF7
Beta
1.00
0.75
0.50
0.25
0.00
56511600
56511650
56511700
56511750
56511800
56511850
56511900
56511950
position (bp)

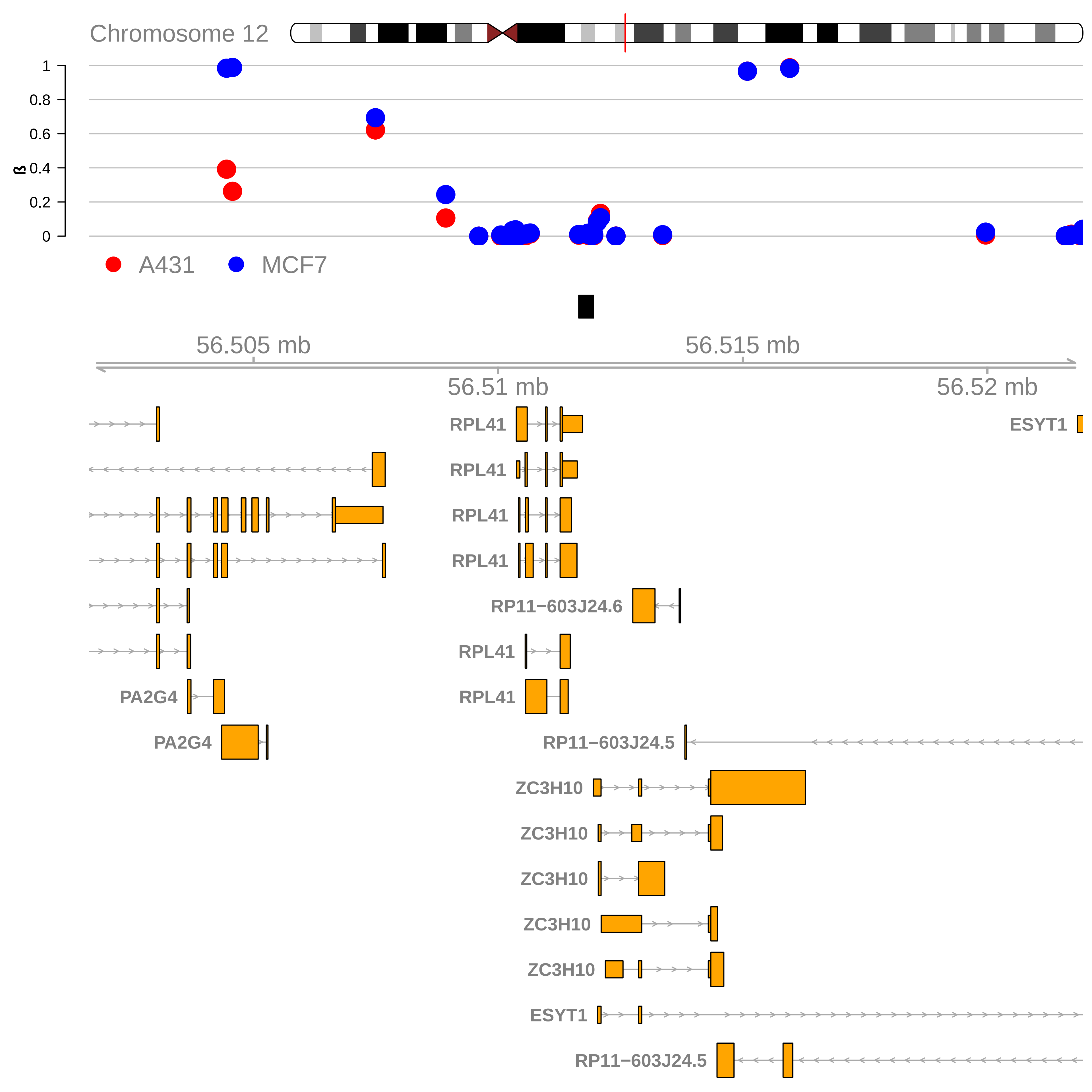

Chromosome 12
1
0.8
0.6
ß 0.4
0.2
0
A431
MCF7
56.505 mb
56.515 mb
56.51 mb
56.52 mb
RPL41
ESYT1
RPL41
RPL41
RPL41
RP11−603J24.6
RPL41
PA2G4
RPL41
PA2G4
RP11−603J24.5
ZC3H10
ZC3H10
ZC3H10
ZC3H10
ZC3H10
ESYT1
RP11−603J24.5
